# Litter quality outweighs climate in driving grassland root decomposition

**DOI:** 10.3389/fpls.2025.1639369

**Published:** 2025-10-01

**Authors:** Jingjing Yang, Zhanbo Yang, Runzhi Zhang, Pingting Guan, Taihai Xu, Yao Tang, Guoling Ren

**Affiliations:** ^1^ School of Biotechnology, Daqing Normal University, Key Laboratory of Applied Chemistry and Technology in Oilfield, Daqing, Heilongjiang, China; ^2^ Key Laboratory of Vegetation Ecology of the Ministry of Education, Jilin Songnen Grassland Ecosystem National Observation and Research Station, Institute of Grassland Science, Northeast Normal University, Changchun, China

**Keywords:** root decomposition, climate, litter quality, meta-analysis, grassland

## Abstract

**Introduction:**

Root decomposition plays a critical role in nutrient cycling and carbon storage in grassland ecosystems, yet its global drivers remain poorly understood.

**Methods:**

The study synthesized global data on root decomposition in grasslands to assess the relative importance of climate and litter quality, and to quantify the effects of environmental and biotic factors using a comprehensive meta-analysis.

**Results:**

Results indicated that, at the global scale, litter quality exerted a stronger influence on root decomposition than climatic variables. Random forest analysis identified the ratio of acid-unhydrolyzable residue to nitrogen (AUR:N) and AUR as the most important predictors of mass loss, both of which were significantly and negatively correlated with mass loss. The meta-analysis further demonstrated that both environmental and biotic factors significantly affected root decomposition. Among environmental factors, nitrogen addition (+4.49%), phosphorus addition (+16.26%), warming (+9.80%), increased precipitation (+5.95%), and elevated CO_2_ (+14.03%) were found to promote root decomposition, while reduced precipitation (−15.60%) had the negative effect. With respect to biotic factors, grazing (+7.51%) significantly increased decomposition, whereas vegetated soil (−27.84%), increased plant species richness (−4.99%), increased root litter richness (−5.93%), home-field decomposition (−4.34%), and soil biota exclusion (−10.40%) decreased it.

**Discussion:**

These findings highlight the dominant role of litter quality over climate in regulating root decomposition at a global scale, and underscore the sensitivity of belowground processes to environmental and biotic disturbances in grassland ecosystems.

## 1 Introduction

Grasslands occupy approximately 52.5 million km², representing about 40.5% of the Earth’s terrestrial surface excluding Greenland and Antarctica, and contribute to roughly 34% of global terrestrial carbon storage (Bai and Cotrufo, 2022; Dondini et al., 2023). Notably, about 90% of this carbon is retained belowground in the form of root biomass and soil organic carbon (Bai and Cotrufo, 2022). Due to their high root-to-shoot ratios, grassland plants allocate a substantial proportion of biomass belowground (Jackson et al., 1997; Yang et al., 2010), providing a major carbon input to the soil (Rasse et al., 2005; Mendez-Millan et al., 2010) and playing a pivotal role in carbon and nutrient cycling (Scheffer and Aerts, 2000).

Litter decomposition is primarily controlled by climate, litter quality and decomposer communities (Coûteaux et al., 1995). At large scales, climate is generally considered the predominant determinant of decomposition rates (Aerts, 1997). Regional climate directly influences decomposition environment (e.g., temperature and moisture regimes), and indirectly alters litter quality by shaping the chemical composition of plant tissues (Suseela and Tharayil, 2018). In contrast, litter quality is often regarded as the most important intrinsic factor controlling decomposition, especially at smaller spatial or experimental scales. A recent meta-analysis demonstrated that the combination of total nutrient (N) content and the C:N ratio explained 70.2% of the variation in litter decomposition rates (Zhang et al., 2008). Moreover, due to its recalcitrant nature, lignin content in litter is frequently identified as a key constraint on both the rate and limit value of decomposition (Berg, 2014). The relative importance of climate and litter quality can also vary depending on the stage of decomposition and the favorability of the environment (Coûteaux et al., 1995; Canessa et al., 2021). However, despite extensive research on the influence of climate and litter quality on aboveground litter decomposition, their regulatory roles in root decomposition, especially within grassland ecosystems, remain poorly understood.

Litter decomposition is generally recognized as a multi-phase process (Berg and McClaugherty, 2020). In the initial stage, easily degradable components such as water-soluble compounds and hemicellulose are rapidly decomposed. Once all unshielded holocellulose has been exhausted, the decomposition process enters a later stage dominated by the degradation of lignified holocellulose and lignin, which proceeds at a substantially slower rate (Berg, 2014). Changes in substrate quality are often accompanied by succession in microbial decomposer communities (Boer et al., 2005). Therefore, any factor that affects either the physical loss or biological degradation of litter can potentially regulate the decomposition process.

In general, nitrogen (N) addition generally stimulates short-term decomposition by enhancing bacterial pathways (Dong et al., 2020; Li et al., 2022b), but may suppress long-term decomposition through inhibition of oxidative enzymes and interactions with acid-unhydrolyzable residues (AUR) (Knorr et al., 2005; Wu et al., 2023). Conversely, phosphorus (P) addition tends to consistently promote decomposition, especially in P-limited grasslands, by alleviating nutrient constraints and balancing N:P stoichiometry (Lu et al., 2022; Wu et al., 2025b). Precipitation influences decomposition through both physical processes, such as leaching, and by affecting decomposer activity (Yahdjian et al., 2006; Krishna and Mohan, 2017). In semiarid grasslands, increased precipitation has been reported to accelerate litter decomposition (Li et al., 2022b). Although some studies have reported that warming enhances litter decomposition (Hobbie, 1996; van Meeteren et al., 2008; Kirwan and Blum, 2011); this pattern is not universal (Walter et al., 2013), and limited mechanistic research prevents firm conclusions.

Livestock grazing can potentially affect belowground decomposition by altering soil microclimate (temperature and moisture) and modifying plant community composition, including root traits (Smith et al., 2014). A global meta-analysis has shown that light grazing strongly promotes litter decomposition (Su et al., 2022b), whereas a study in the Inner Mongolian grasslands reported that grazing inhibited the mass loss rate of root litter, a pattern mediated by changes in the microbial biomass carbon-to-nitrogen ratio (Li et al., 2022a). Aboveground vegetation also regulates root decomposition, either positively—through the release of root exudates that stimulate organic matter breakdown—or negatively, by diverting microbial activity away from litter decomposition (Kaštovská et al., 2015; Yin et al., 2021; Heredia-Acuña et al., 2023; Zheng et al., 2023). Increasing plant species richness can increase the quantity of root exudates and reshape soil microbial communities (Eisenhauer et al., 2017); by influencing mycorrhizal fungi, further modulate saprotrophic fungal activity, with likely implications for root decomposition (Choreño-Parra and Treseder, 2024; van Galen et al., 2025), but empirical evidence remains limited. Although microorganisms are the primary agents of litter decomposition, soil fauna can further accelerate the process (Li et al., 2024b), both directly through fragmentation and ingestion of litter (Kaneda et al., 2013; Frouz et al., 2015), and indirectly by modifying microbial community composition and activity (Frouz, 2018; Huang et al., 2020). Therefore, understanding how multiple environmental and biotic factors regulate grassland root decomposition at the global scale is essential for improving predictions of belowground carbon and nutrient cycling.

In this study, we used global data on grassland root decomposition to assess the relative importance of climate and litter quality at the global scale. We further conducted a comprehensive global meta-analysis to evaluate the effects of environmental and biotic factors—defined here as regulatory drivers associated with animals, plants, and litter—on root decomposition in grasslands. Based on current knowledge, we proposed the following hypotheses: (1) Litter quality exerts a stronger influence on root decomposition than climate, particularly during the later stages of decomposition, due to the increasing role of recalcitrant compounds. (2) Nutrient additions (N and P), warming, and increased precipitation are generally expected to promote root decomposition by alleviating nutrient limitations and enhancing microbial activity, whereas reduced precipitation is predicted to inhibit decomposition. (3) Biotic factors such as grazing and plant species richness affect root decomposition, with differences in their direction and magnitude.

## 2 Materials and methods

### 2.1 Data compilation

We systematically searched the Web of Science (https://apps.webofknowledge.com) and China National Knowledge Infrastructure (https://www.cnki.net) databases for peer-reviewed publications published from 1985 to March 2025. The search strategy employed the following terms: TS = (“grassland*” OR “prairie” OR “savanna” OR “steppe” OR “pampas”) AND TS = (“degrad*” OR “breakdown” OR “decomp*”) AND TS = (“root*” OR “belowground”), targeting studies relevant to root decomposition in grassland ecosystems (**Supplementary Figure S1**). Studies were included based on the following criteria: (1) the study was conducted in grassland ecosystems under natural environmental conditions (with the exception of one pot experiment, which was included only in the meta-analysis of the vegetated soil factor); (2) control and treatment groups were implemented at the same site and during the same time period; (3) the decomposition substrate consisted exclusively of grassland plant roots, excluding studies focusing on belowground decomposition of aboveground plant parts or other substrates; and (4) the study reported either litter mass loss or litter decomposition rate constants (k), along with decomposition duration, derived from text, figures, or tables. Data on latitude, longitude, mean annual temperature (MAT), and mean annual precipitation (MAP) were obtained directly from the original articles or inferred from other studies conducted at the same sites. Missing elevation data were extracted using Google Earth (https://earth.google.com/) based on the reported geographic coordinates. For studies reporting only decomposition rate constants (k), litter mass loss was recalculated using established equations. Where neither standard deviation (SD) nor standard error (SE) was reported, SD was approximated as one-tenth of the mean (Luo et al., 2006). If variability was reported but it was unclear whether it referred to SD or SE, we assumed it was SE and converted it to SD accordingly (Treseder, 2004).

Based on the above criteria, a total of 73 articles were included in the analysis. Among these, 1,360 observations were used in the global Random Forest analysis, representing litter mass loss at each time point for each type of root litter under natural environmental conditions. Additionally, 1,127 observations were included in the meta-analysis, each corresponding to the mass loss of root litter at a given time point for a pair of treatment and control conditions. We extracted mass loss data at each sampling point from each study (restricted to non-replacement sampling) and retained decomposition duration as a variable, rather than using or calculating a decomposition rate constant (k). This is because different litter components decompose at varying rates, and decomposition slows significantly in later stages due to the accumulation of recalcitrant compounds (Wider and Lang, 1982). As a result, longer decomposition durations tend to yield smaller k values, making k an unsuitable basis for cross-study comparisons.

In addition to duration, we extracted the following variables from each study: root burial depth, root diameter, litterbag mesh size, latitude, longitude, MAT, MAP, elevation, and initial root litter chemistry, including AUR, total carbon (C), total nitrogen (N), AUR:N ratio, and C:N ratio. The data used was collected from the original published articles. When numerical data were not directly available in tables or text, values were extracted from published figures using the digital digitizing tool in OriginLab 2025.

### 2.2 Statistical analyses

**Figure 1:**
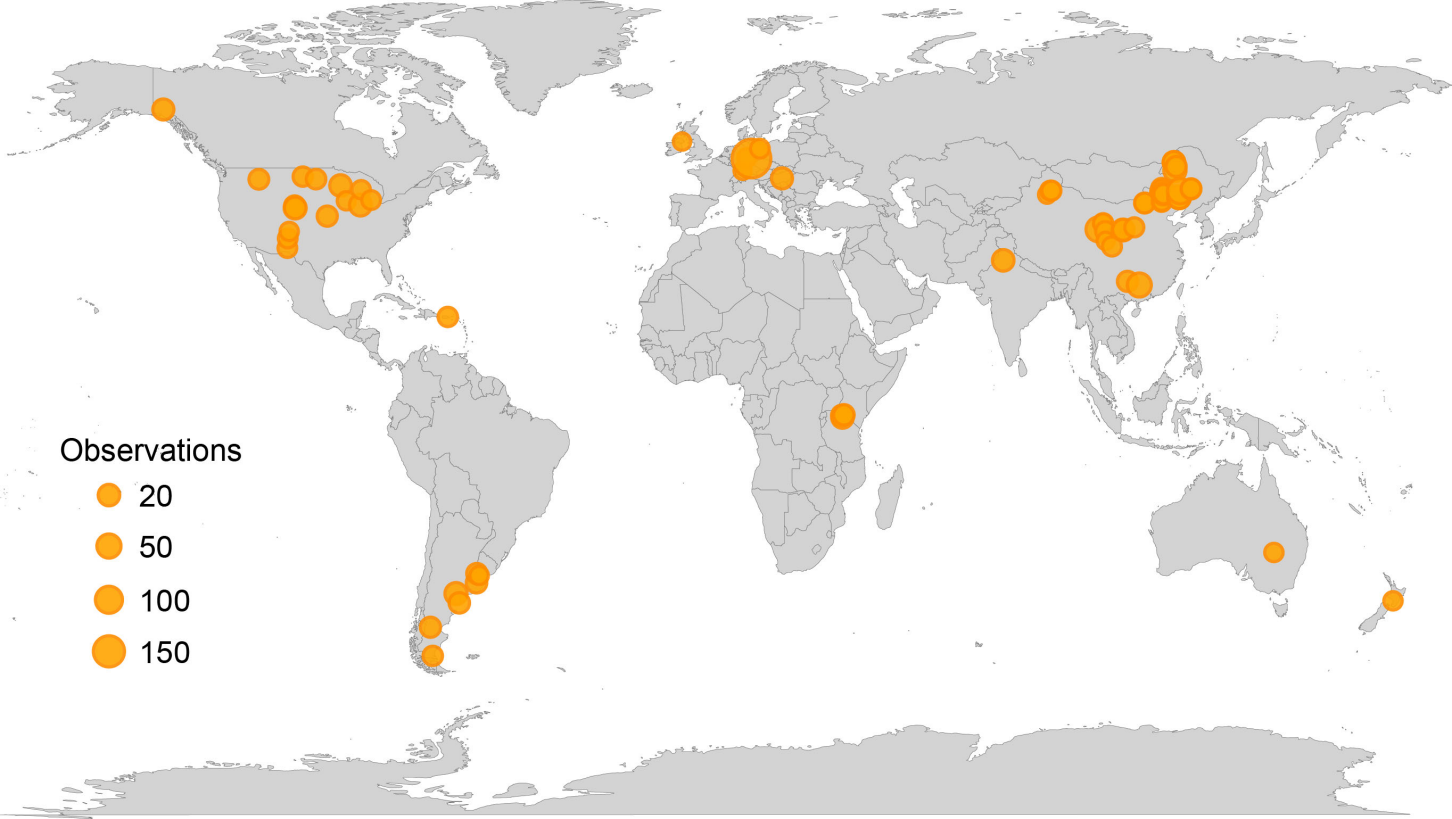
Global distribution of study sites used in the Random Forest analysis. All sites represent control groups either from natural ecosystems or from field experiments, reflecting conditions without experimental manipulation. The size of the triangles represents the number of observations at each site.

The random forest model was based on decomposition data collected from natural grassland sites or experimental sites influenced solely by ambient environmental conditions, including control groups from manipulation experiments. In total, the dataset encompassed 69 grassland sites distributed globally (**Figure 1**). Lignin content was considered in the model only for studies that used the acid-unhydrolyzable residue (AUR) method as an indicator of lignin. Random forest modeling and significance testing were conducted in R using the “rfPermute” package. The individual effects of litter quality and geoclimatic factors were quantified using hierarchical partitioning analysis with the “glmm.hp” package, with PCA-derived indices (PC1) employed to represent composite measures of litter quality and geoclimatic factors.

Meta-analysis was conducted to assess the effects of environmental factors (nitrogen addition, phosphorus addition, warming, increased precipitation, reduced precipitation, and elevated CO_2_) and biotic factors (grazing, vegetated soil, elevated plant richness, elevated litter richness, home-field decomposition, and soil biota exclusion) on mass loss during root decomposition in grasslands. The natural logarithm of the response ratio (log_e_RR) was used to quantify effect sizes (Equation 1), along with the calculation of variance (v) (Equation 2) and weighting factor (w) (Equation 3), following the formulas below:


(1) $$\begin{array}{c} {\text{log}}_{\text{e}}\text{RR}={\text{log}}_{\text{e}}(\frac{{\text{X}}_{\text{t}}}{{\text{X}}_{\text{c}}})={\text{log}}_{\text{e}}\left({\text{X}}_{\text{t}}\right)-{\text{log}}_{\text{e}}({\text{X}}_{\text{c}}) \end{array}$$

(2) $$\begin{array}{c} v=\frac{{\text{S}}_{\text{t}}^{2}}{{\text{n}}_{\text{t}}{\text{X}}_{\text{t}}^{2}}+\frac{{\text{S}}_{\text{c}}^{2}}{{\text{n}}_{\text{c}}{\text{X}}_{\text{c}}^{2}} \end{array}$$

(3) $$\begin{array}{c} w=\frac{1}{\text{v}} \end{array}$$

where X_t_, S_t_, and n_t_ represent the mean values of mass loss rate, standard deviation, and sample size for the treatment group, respectively, while X_c_, S_c_, and n_c_ represent the corresponding values for the control group.

The log_e_RR values were assumed to follow a normal distribution and were fitted with a Gaussian model (Lu et al., 2011). A fixed-effects model was initially used to calculate the global (mean) effect size (RR_++_). If the test for total heterogeneity was significant, a mixed-effects (random-effects) model was subsequently applied to recalculate RR_++_. The percentage change in mass loss under each factor was estimated by ( ${\text{e}}^{{\text{RR}}_{++}}-1)\times 100\%$ . Grouped meta-analyses were conducted using duration as a categorical moderator, ensuring that each subgroup included data from at least 3 independent studies or a minimum of 10 observations (Wittig et al., 2009; Feng et al., 2010). Meta-regression analyses were performed to examine how initial litter chemical traits (litter quality) and geoclimatic factors influenced the effect sizes of various environmental and biotic factors on root decomposition.

Statistical significance was assessed using confidence intervals based on resampling methods (64999 iterations) (Dieleman et al., 2010). Confidence intervals based on bootstrapping tests are wider than standard confidence intervals, implying that resampling estimates are more conservative (Adams et al., 1997). An effect was considered statistically significant if the bias-corrected bootstrap confidence interval did not include zero. All meta-analytical procedures, including the calculation and pooling of effect sizes, were conducted using MetaWin 3.0 (Rosenberg, 2024). Our data were assessed for publication bias using fail-safe numbers, with all meta-analyses meeting the threshold of 5n + 10 (where n is the number of observations) (Rosenberg, 2005).

## 3 Result

### 3.1 Relative importance of geoclimatic factors and litter quality in explaining root decomposition

**Figure 2:**
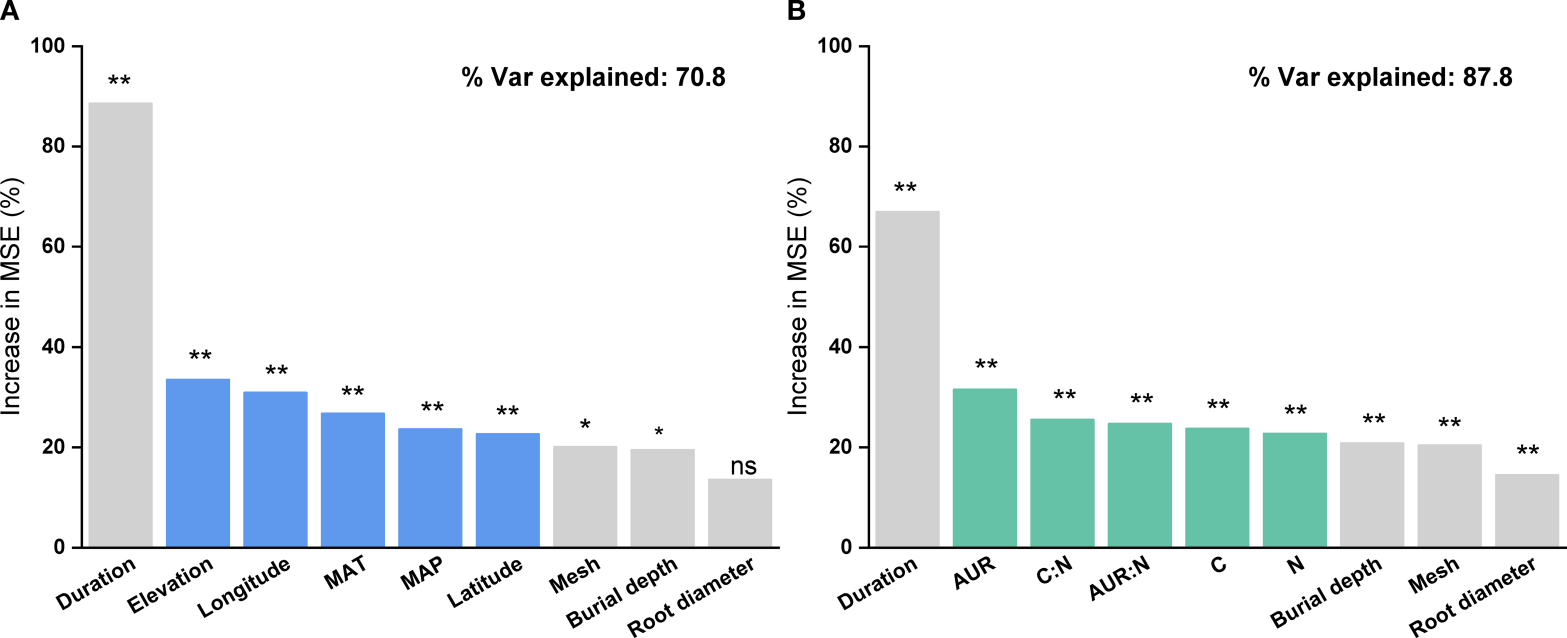
Relative importance of **(A)** geoclimatic factors only (latitude, longitude, elevation, mean annual temperature (MAT), and mean annual precipitation (MAP)) and **(B)** initial root litter chemistry only (acid-unhydrolyzable residue (AUR), C, N, P, AUR:N, and C:N) in explaining variation in root litter decomposition, based on Random Forest analysis. **P < 0.01; *P < 0.05; ns, not significant.

**Figure 3:**
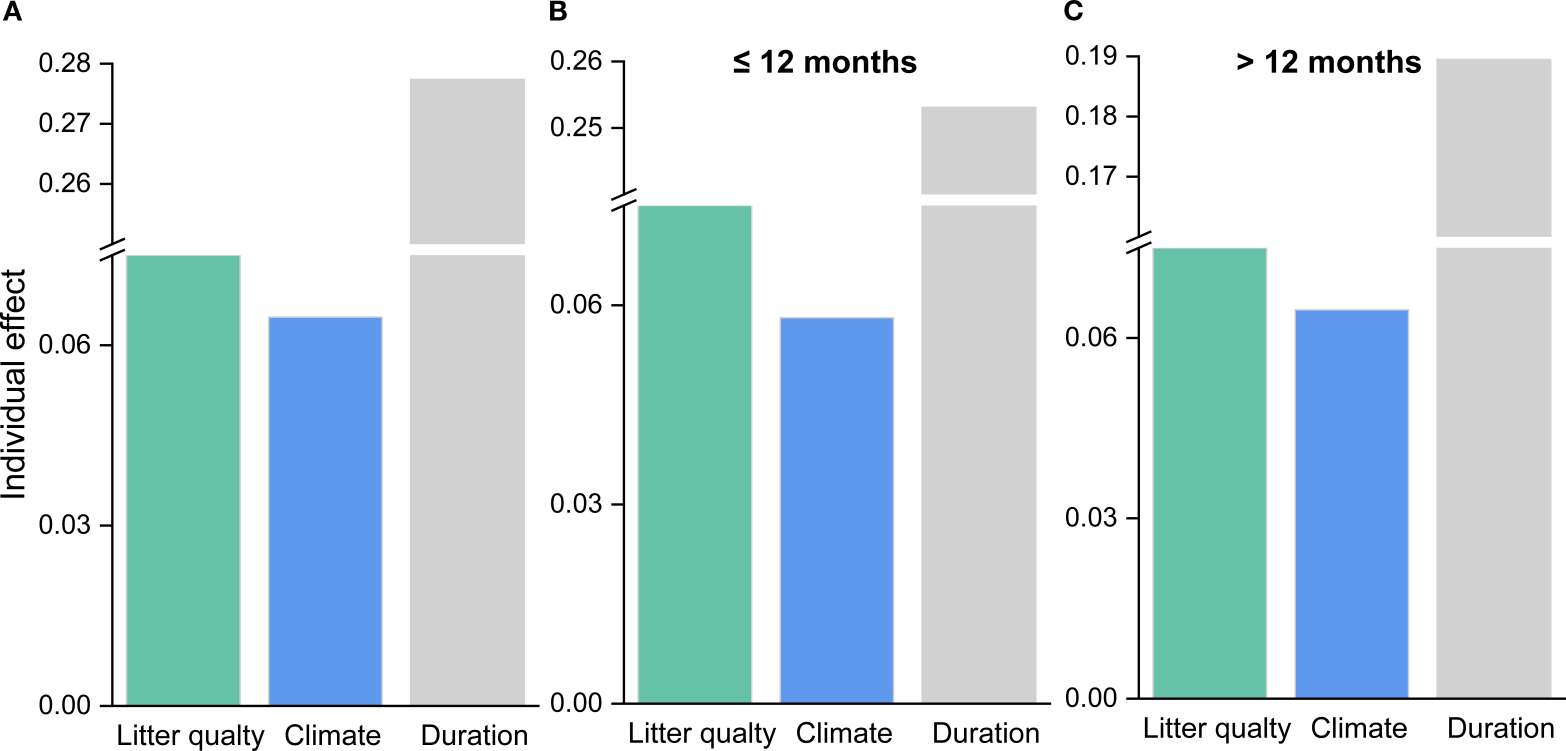
Individual importance of predictor variables in explaining the residuals of mass loss for **(A)** all data, **(B)** data with decomposition time ≤ 12 months, and **(C)** data with decomposition time > 12 months.

**Figure 4:**
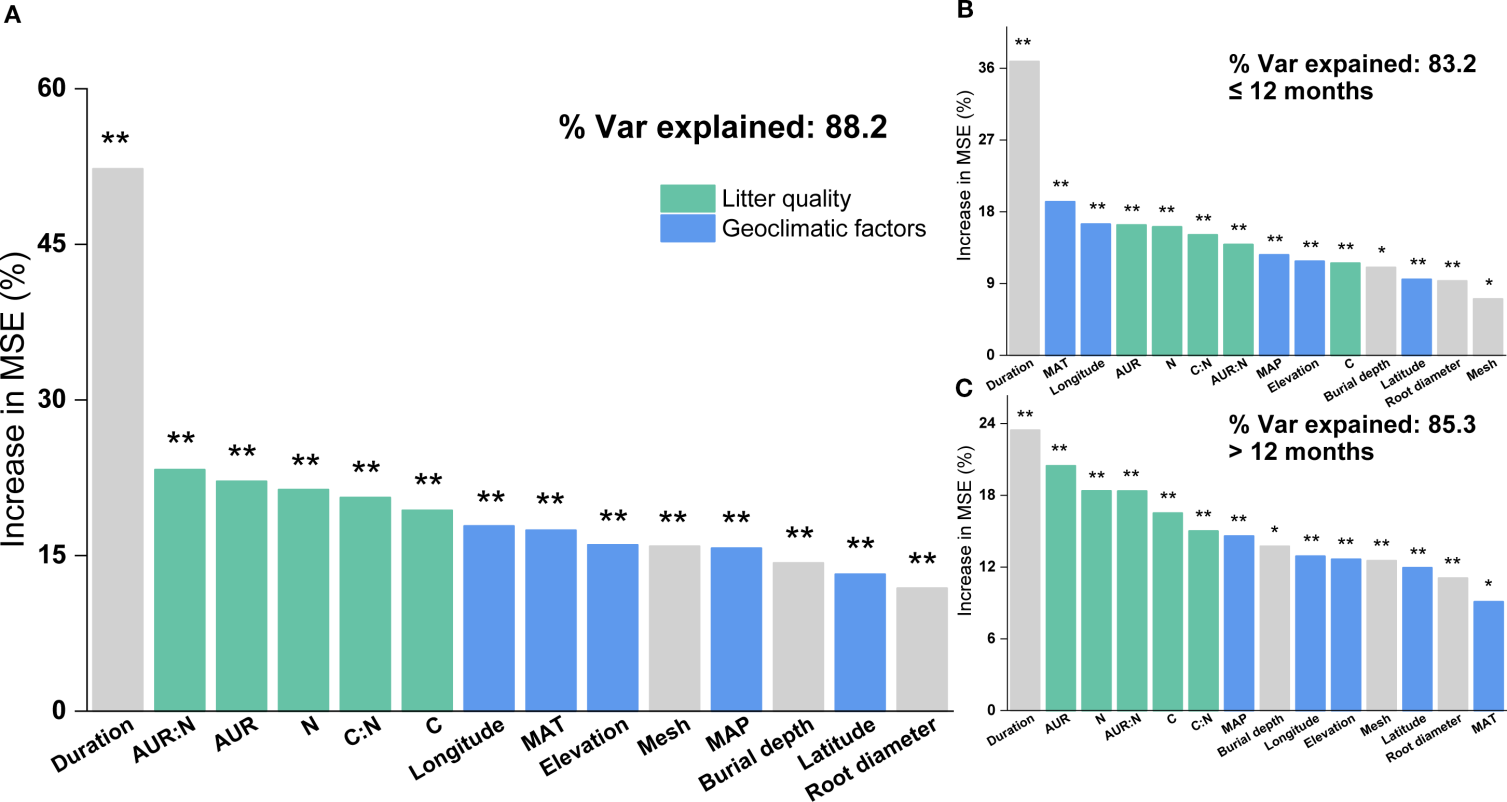
Relative importance of geoclimatic factors (latitude, longitude, elevation, mean annual temperature (MAT), and mean annual precipitation (MAP)) and initial root litter chemistry (acid-unhydrolyzable residue (AUR), C, N, P, AUR: N, and C: N) in explaining variation in root litter decomposition, based on Random Forest analysis. **(A)** All data combined; **(B)** decomposition within 0–12 months; **(C)** decomposition after 12 months. **P <0.01; *P <0.05.

**Figure 5:**
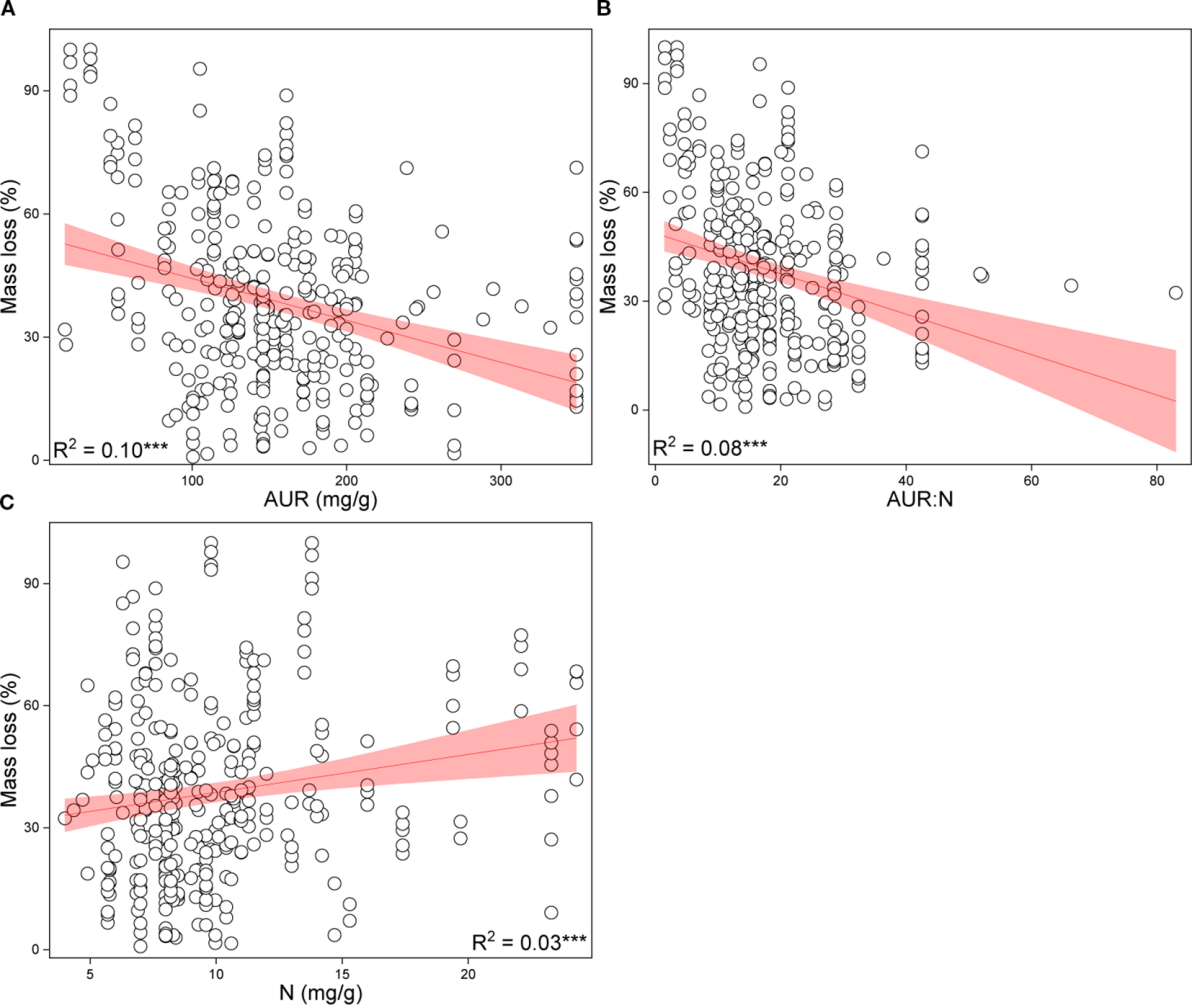
Correlations between root mass loss and acid-unhydrolyzable residue (AUR) **(A)**, AUR:N ratio **(B)**, and nitrogen (N) content **(C)**. ***P <0.001.

Based on the random forest results, models using only geoclimatic variables explained 70.8% of the variation in grassland root decomposition at the global scale, whereas models based solely on root litter quality accounted for 87.8% (**Figure 2**). Thus, litter quality provided stronger explanatory power than geoclimatic factors, consistent with results from hierarchical partitioning analysis (**Figures 2**, **3**). When both geoclimatic variables and litter quality were included, the model explained 88.2% of the variation, with a higher mean contribution from litter quality (21.34%) than from geoclimatic variables (16.03%) (**Figure 4A**). Temporal partitioning of the data using 12 months as the threshold revealed a shift in the relative importance of predictors: during the early stage of decomposition (≤12 months), both geoclimatic factors and litter quality played important roles in driving root decomposition (**Figure 4B**). In contrast, in the later stage (>12 months), litter quality played a more dominant role, contributing 17.73% on average compared to 12.23% from geoclimatic variables (**Figure 4C**). Moreover, both AUR:N and AUR were significantly negatively correlated with mass loss, whereas N showed a significant positive correlation with mass loss (**Figure 5**).

### 3.2 Meta-analysis of root litter decomposition responses to environmental and biotic factors

**Figure 6:**
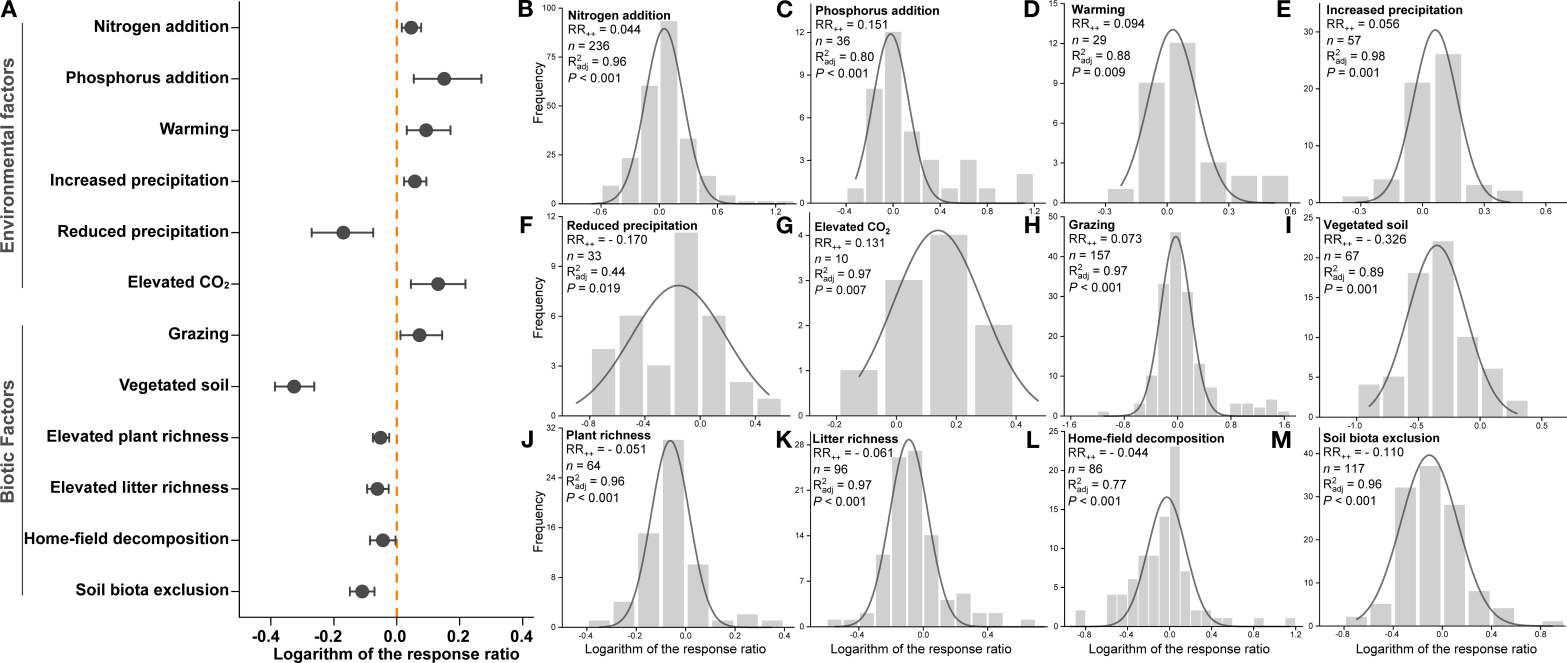
The weighted response ratio (RR_++_) for the effects of twelve environmental and biotic factors on root litter decomposition **(A)**, and the frequency distributions of the natural logarithm of the response ratio (log_e_RR) for each individual factor: nitrogen addition **(B)**, phosphorus addition **(C)**, warming **(D)**, increased precipitation **(E)**, reduced precipitation **(F)**, elevated CO_2_ **(G)**, grazing **(H)**, vegetated soil **(I)**, elevated plant richness **(J)**, elevated litter richness **(K)**, home-field decomposition **(L)**, and soil biota exclusion **(M)**. The solid curves represent Gaussian distributions fitted to the frequency data. The x-axis denotes log_e_RR, and the y-axis denotes frequency.

Meta-analysis results showed that both environmental and biotic factors had significant effects on root litter decomposition, with confidence intervals excluding zero, and the effects differed significantly between these two groups (**Figure 6A**).

Among environmental factors, only reduced precipitation caused a significant decrease in decomposition rate (−15.60%) (**Figure 6F**), while nitrogen addition (+4.49%) (**Figure 6B**), phosphorus addition (+16.26%) (**Figure 6C**), warming (+9.80%) (**Figure 6D**), increased precipitation (+5.95%) (**Figure 6E**), and elevated CO_2_ (+14.03%) (**Figure 6G**) caused significant increases.

For biotic factors, grazing was the only factor associated with a significant increase in decomposition (+7.51%) (**Figure 6H**), vegetated soil (−27.84%) (**Figure 6I**), increased plant species richness (−4.99%) (**Figure 6J**), increased root litter richness (−5.93%) (**Figure 6K**), home-field decomposition (−4.34%) (**Figure 6L**), and soil biota exclusion (−10.40%) (**Figure 6M**) caused significant decreases.

### 3.3 Temporal variation in the effects of environmental and biotic factors on root litter decomposition

Meta-regression analysis revealed significant time-dependent effects for nitrogen addition (*P*<0.05), vegetated soil (*P*<0.001), increased plant species richness (*P*<0.001), increased root litter richness (*P*<0.01), and soil biota exclusion (*P*<0.001) (**Supplementary Table S1**).

**Figure 7:**
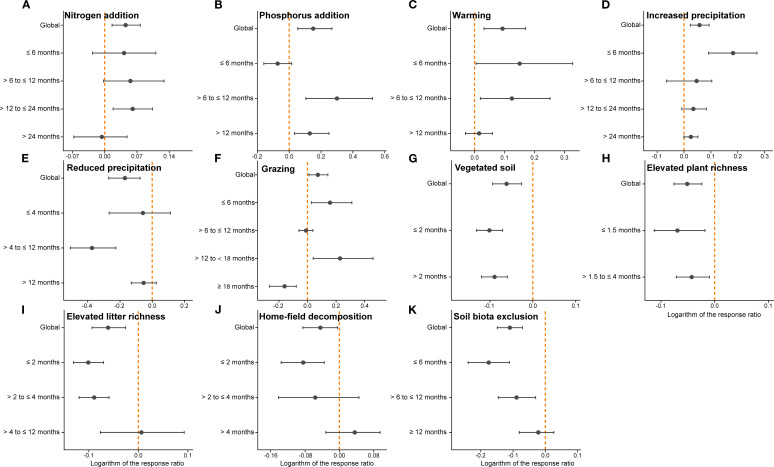
The weighted response ratio (RR_++_) for the effects of eleven individual environmental and biotic factors on root litter decomposition, grouped by decomposition duration. Each panel (a–k) shows the temporal dynamics of treatment effects for nitrogen addition **(A)**, phosphorus addition **(B)**, warming **(C)**, increased precipitation **(D)**, reduced precipitation **(E)**, grazing **(F)**, vegetated soil **(G)**, elevated plant richness **(H)**, elevated litter richness **(I)**, home-field decomposition **(J)**, and soil biota exclusion **(K)**. Error bars represent bias-corrected bootstrap (64999) confidence intervals. The vertical dashed line in orange indicates log_e_RR = 0.

Time-grouped meta-analyses further showed that the effects of most environmental and biotic factors varied across decomposition stages (**Figure 7**). N addition had generally positive effects during the early phase but tended to shift toward negative values after 24 months (**Figure 7A**). P addition exhibited a significant positive effect after 6 months of decomposition (**Figure 7B**). Warming initially stimulated decomposition, but its effect declined over time and became non-significant for root decomposition by 12 months (**Figure 7C**). Increased precipitation significantly enhanced decomposition during the first 6 months; beyond this period its positive effect was not statistically significant (**Figure 7D**). In contrast, reduced precipitation consistently suppressed root decomposition, with a significant negative impact observed between 4–12 months (**Figure 7E**).

Grazing promoted decomposition prior to 18 months, but its effect turned negative afterward (**Figure 7F**). The effects of vegetated soil and increased plant species richness showed little temporal change (**Figures 7G, H**), possibly due to the generally short decomposition durations in these studies. Increased litter richness significantly reduced decomposition before 4 months, but had no clear effect at longer durations (**Figure 7I**). The negative effects of home-field decomposition and soil biota exclusion weakened over time, with a trend toward neutral or even positive effects at later stages (**Figures 7J, K**).

## 4 Discussion

### 4.1 Effects of climate and litter quality on grassland root decomposition

Consistent with our first hypothesis and previous studies, our analysis shows that when climate and litter quality are evaluated in the same model, litter quality explains decomposition rates more effectively than climate (**Figures 3**, **4**) (Zhang et al., 2008; Waring, 2012; Djukic et al., 2018). Even in a global, multi-ecosystem analysis of root decomposition patterns, litter quality exhibited a stronger explanatory power than climatic variables (Silver and Miya, 2001). This may be attributed to the different mechanisms through which climate and litter quality influence decomposition.

Initial litter quality exerts a persistent influence throughout the decomposition process, with N content and AUR content being particularly critical (**Figure 4**). Specifically, AUR content is negatively correlated with mass loss, whereas N content is positively correlated with mass loss (**Figure 5**). Higher initial N content generally accelerates decomposition by alleviating N limitation over the course of the decomposition process (Wu et al., 2023), possibly by stimulating the activity of N- and oxidative-enzymes and enhancing the overall microbial capacity for degradation (Talbot and Treseder, 2012; Yang et al., 2025). In contrast, AUR content represents a major rate-limiting component of litter decomposition, as its breakdown can be efficiently mediated only by oxidative enzymes and certain specialized fungal taxa (Eastwood et al., 2011). This AUR “barrier effect” also restricts the accessibility and degradation of more labile polysaccharides such as cellulose and hemicellulose (Berg and McClaugherty, 2020). Moreover, AUR degradation products may interact with N compounds to form more recalcitrant complexes, further slowing decomposition (Berg, 2014).

In climatic analyses, MAP and MAT are commonly used as proxies for climate conditions. However, these metrics may have limited explanatory power for decomposition processes, as they fail to capture key dynamics such as precipitation frequency and seasonal variation in temperature and moisture. Indeed, studies have shown that precipitation or temperature fluctuations during specific decomposition stages can exert disproportionately strong effects on decomposition (Fierer et al., 2005; Anaya et al., 2012; Joly et al., 2017). Moreover, soil microbial communities often exhibit a degree of resistance to environmental change (Jiao et al., 2022b). In highly diverse communities, functional redundancy ensures that the stability of core microbial taxa can maintain overall functional performance (Jiao et al., 2022a), which may contribute to a certain resilience of microbially mediated root decomposition to climate change.

### 4.2 Responses of grassland root decomposition to environmental factors

A meta-analysis showed that the effect of N addition on litter decomposition closely depends on litter quality, promoting decomposition of high-quality litter but inhibiting that of low-quality litter (Knorr et al., 2005). In grassland ecosystems, N addition generally tends to increase decomposition, which is likely attributable to the relatively low lignin content in grassland litter and the comparatively weak dominance of Basidiomycota—the primary fungal group responsible for oxidative enzyme production (Dong et al., 2020). Mechanistically, N inputs first enhance the activity of carbohydrate-degrading enzymes, directly facilitating the degradation of cellulose and hemicellulose (Dong et al., 2022). Additionally, N-induced decreases in soil pH can increase manganese availability and the bacteria-to-fungi ratio, further promoting decomposition (Hou et al., 2021). However, as decomposition progresses and litter quality declines, lignin increasingly governs decomposition rates (Berg, 2014). Under these conditions, the positive effects of N addition diminish or even become inhibitory (Gill et al., 2022), mainly due to the suppressive effects of N on oxidative enzymes, which limits lignin breakdown (Jian et al., 2016). Grassland ecosystems commonly experience P limitation (Du et al., 2020; Hou et al., 2020), which is further exacerbated under the global context of increased N deposition (Wang et al., 2025). In this scenario, exogenous P inputs often enhance soil microbial activity (Lu et al., 2022), potentially promoting the decomposition of grassland litter. Such enhancement is exemplified by a study in a northern temperate grassland, where P addition was shown to stimulate hydrolytic and oxidative enzyme activities, thereby promoting litter decomposition (Shi et al., 2021).

Warming can affect decomposition by altering microbial and enzymatic activities (Allison et al., 2010). A meta-analysis reported that warming significantly increased litter decomposition by 4.4% (Yue et al., 2015), consistent with our findings (**Figure 6D**). Overall, warming can enhance soil enzyme activities to varying degrees, which facilitates the mineralization and decomposition of soil organic matter (Meng et al., 2020; Zuccarini et al., 2020; Fanin et al., 2022). Notably, the effect of warming tends to diminish over time, potentially due to the weak temperature sensitivity of lignin degradation (**Figure 7C**) (Liu et al., 2021). Precipitation affects decomposition through both physical processes, such as leaching, and by regulating microbial activity. In general, increased precipitation promotes decomposition, whereas reduced precipitation inhibits it (**Figures 6E, F**), with the sizes of these effects closely related to site aridity and rainfall levels (Su et al., 2023; Liu et al., 2024). Improved moisture conditions enhance both the abundance and activity of soil microorganisms (Huang et al., 2015), promoting a relative increase in fungal dominance (Cregger et al., 2012; Yang et al., 2021), which contributes to the degradation of recalcitrant compounds. Increased precipitation has also been shown to elevate soil N- and P-acquiring enzyme activities (Li et al., 2024a), which can facilitate the decomposition of soil organic matter. Unlike previous multi-ecosystem meta-analyses (Wu et al., 2025a), we observed a positive effect of elevated CO_2_ on root decomposition in grasslands. This may be related to the stimulation of soil enzyme activities under elevated CO_2_ conditions in grasslands (Ebersberger et al., 2003; Kandeler et al., 2006); however, direct evidence remains limited, and further research in this area is needed.

Notably, environmental factors on decomposition can be additive or antagonistic (**Supplementary Figure S2**), and are further modulated by litter quality (**Supplementary Table S2**) (Zhao et al., 2024). Moreover, results derived solely from decomposition experiments may underestimate the influence of environmental drivers, as plant chemical composition is already shaped by environmental conditions prior to senescence, ultimately determining litter quality (Suseela and Tharayil, 2018). Future studies should consider how environmental factors influence both litter quality and decomposition processes, as well as their interactions, to enhance our understanding and predictive capacity regarding grassland ecosystem functioning under global change.

Overall, nutrient additions and improvements in hydrothermal conditions promoted root decomposition, consistent with our second hypothesis. Under the influence of global change and human activities, grassland ecosystems are experiencing shifts in nutrient inputs and environmental conditions (Su et al., 2022a; Liu et al., 2023). These changes profoundly impact soil carbon dynamics and nutrient cycling by regulating root decomposition. Our results reveal how grassland root decomposition responds to multiple environmental factors, highlighting its critical role in predicting belowground ecosystem dynamics.

### 4.3 Responses of grassland root decomposition to biotic factors

Consistent with our third hypothesis, multiple biotic factors can influence root decomposition, but their effects are not always in the same direction. Previous meta-analyses have shown that grazing on average promotes root decomposition, particularly in grassland ecosystems (**Figure 6H**) (Su et al., 2022b; Jiang et al., 2024). Compared with aboveground litter, grazing has little effect on root decomposition through physical fragmentation. But it can still alter plant community composition, root traits and exudates, as well as microbial community structure and biomass, thereby creating soil resource and biotic conditions that are more favorable for root decomposition (Klumpp et al., 2009; Wilson et al., 2018). Similarly, Tan et al. (2024) reported that grazing accelerates soil organic carbon turnover. Although grazing has been shown to reduce the activity of multiple soil enzymes (Olivera et al., 2014; Feng et al., 2025), evidence suggests that it enhances microbial growth and fungal dominance, leading to improved carbon use efficiency and greater utilization of soil organic matter, rather than relying solely on enzyme activity (Zhang et al., 2024). Additionally, the effects of grazing on decomposition varied across stages, which may be related to litter quality (**Supplementary Table S2**). Specifically, grazing promoted the degradation of hemicellulose and cellulose but had limited effects on lignin (Su et al., 2022b), resulting in a positive effect primarily during holocellulose-dominated stages.

Plant cover (i.e., vegetated soil) and increased plant richness both suppressed root decomposition, and the negative effect of plant richness increased with richness (**Figures 6I, J**, **Supplementary Table S2**). On the one hand, the presence of living roots can alter soil microbial communities through root exudates, directing microbial activity toward utilizing exudates rather than participating in decomposition (Heredia-Acuña et al., 2023). On the other hand, competition between plants and microbes for soil nutrients may limit microbial decomposition. Higher plant richness typically enhances competitive ability and further modifies microbial community composition (Schlatter et al., 2015), resulting in a community less specialized for decomposition.

Overall, mixed-root decomposition exhibited antagonistic rather than additive effects (**Figure 6K**), and the antagonistic effect increased with litter richness (**Supplementary Table S2**). Based on the currently limited evidence, interactions among litter chemical components may play a role (Heredia-Acuña et al., 2023), potentially dependent on species identity (Wu et al., 2013) and environmental context (Porre et al., 2020). Further research is needed to substantiate these effects. Consistent with many previous studies (Pastorelli et al., 2021; van den Brink et al., 2023), our comparison of decomposition in “home” versus “away” environments revealed no evidence of a home-field advantage (**Figure 6L**). This phenomenon remains highly debated; however, it is clear that litter quality and the general ability of the decomposer community influenced litter decomposition much more strongly than origin or location of the litter (Makkonen et al., 2012; Pugnaire et al., 2023). Therefore, future studies on home-field advantage should focus more on these factors rather than on litter origin alone.

Both globally and regionally, soil fauna generally exert positive effects on litter mass loss (García-Palacios et al., 2013). Through litter consumption, fragmentation, and modulation of microbial decomposer communities, soil fauna actively participate in the decomposition process (Angst et al., 2024). Consequently, their exclusion typically leads to reduced decomposition rates (**Figure 6M**). Soil fauna can consume large proportions of annual litter production, assimilating part of the ingested material and returning the remainder to the soil as fecal matter (Frouz, 2018). The passage of litter through the digestive tract causes fragmentation (Gunnarsson et al., 1988; Kaneda et al., 2013), thereby increasing its surface area and potentially enhancing microbial contact with the litter (Cao et al., 2024). Moreover, by feeding on microorganisms, soil fauna accelerate microbial biomass turnover (Bonkowski et al., 2000; Frouz and Nováková, 2001; Crowther et al., 2011), helping to sustain microbial activity (van der Drift and Jansen, 1977; Frouz et al., 2003). Notably, the magnitude of soil fauna effects is also influenced by litter quality and climatic conditions (**Supplementary Tables S1**, **S2**) (Wall et al., 2008; García-Palacios et al., 2013; Xu et al., 2020).

Grasslands worldwide are undergoing varying degrees of degradation, leading to shifts in plant communities and soil biota (Bardgett et al., 2021). These changes exert complex effects on root decomposition, as reflected in our results showing both promotion and inhibition. The differential impacts of these factors on root decomposition are closely linked to soil microbial community and enzyme activities (Heredia-Acuña et al., 2023). Therefore, to improve the accuracy of models predicting soil carbon dynamics in grasslands, it is essential not only to incorporate microbial variables but also to consider other biotic factors such as soil fauna and plant community characteristics (Bradford et al., 2007). This integrated approach will better capture the multifaceted biological controls underlying root decomposition in changing grassland ecosystems.

## 5 Conclusions

The study presents a comprehensive global-scale assessment of the patterns and drivers of grassland root decomposition. At broader spatial scales, litter quality—rather than climate—emerged as the dominant factor influencing root decomposition. In addition, meta-analyses have found that nutrient additions and improved hydrothermal conditions both contribute to increased root mass loss to varying degrees. Biotic factors, such as livestock grazing and plant diversity, also significantly influence root mass loss; however, the direction of their effects is inconsistent, likely reflecting differences in their regulatory mechanisms on soil microbial communities. These findings highlight the high sensitivity of belowground decomposition processes to environmental change in grassland ecosystems. To better understand grassland ecosystem functioning under global change, future research should prioritize long-term, multifactorial experiments on root decomposition.

## Data Availability

The raw data supporting the conclusions of this article are available from the corresponding author upon reasonable request.
