# Supplementary material for: Litter quality outweighs climate in driving grassland root decomposition
Source: Front Plant Sci. 2025 Oct 1;16:1639369. doi: 10.3389/fpls.2025.1639369 (PMC12521445; doi:10.3389/fpls.2025.1639369)
Supplement: Supplementary file 1 [file Table1.docx]

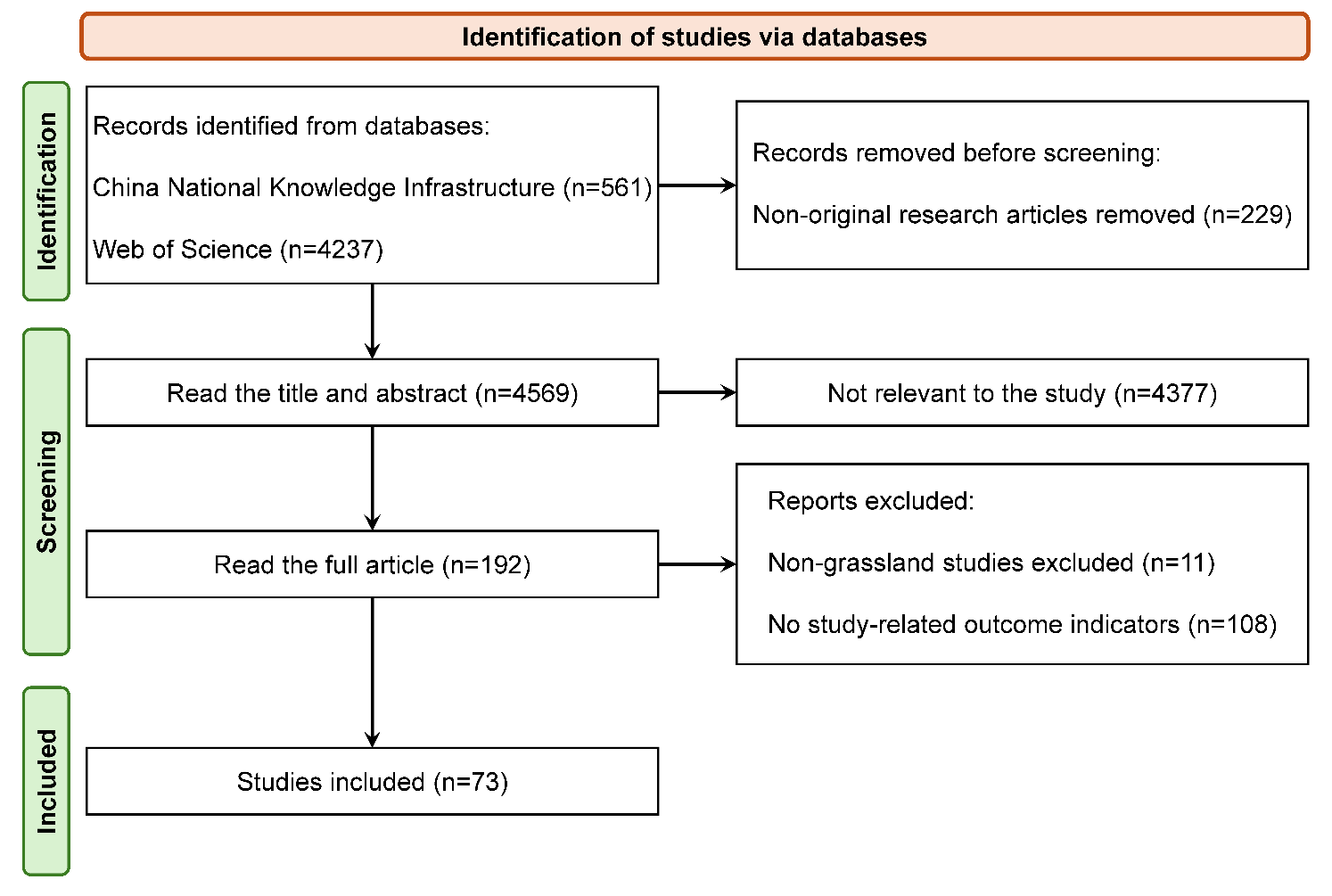


**Supplementary Figure S1** Searching and filtering flowcharts.


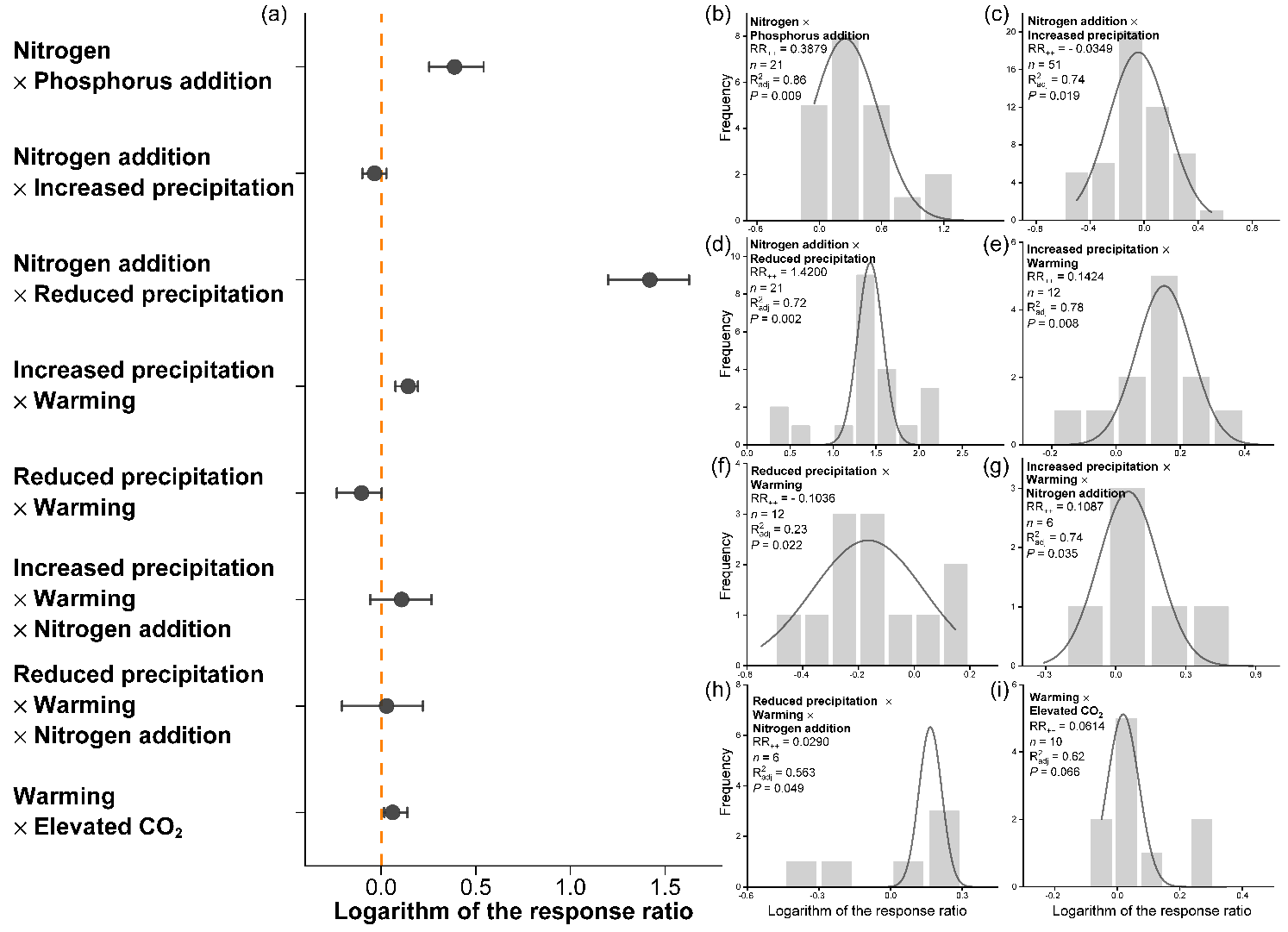


**Supplementary Figure S2** The weighted response ratio (RR_++_) for the effects of multiple environmental factor interactions on root litter decomposition (a), and the frequency distributions of the natural logarithm of the response ratio (log_e_RR) for individual interaction terms: nitrogen × phosphorus addition (b), nitrogen addition × increased precipitation (c), nitrogen addition × reduced precipitation (d), increased precipitation × warming (e), reduced precipitation × warming (f), increased precipitation × warming × nitrogen addition (g), reduced precipitation × warming × nitrogen addition (h), and warming × elevated CO₂ (i). Solid curves represent Gaussian distributions fitted to the frequency data. The x-axis denotes log_e_RR, and the y-axis denotes frequency.

**Supplementary Table S1** Meta-regression analyses assessing the moderating effects of decomposition duration and geoclimatic variables on the effect sizes (logeRR) of root litter decomposition in response to environmental and biotic factors.

| **Treatment effects** | **Duration** | **Longitude** | **Latitude** | **Elevation** | **MAP** | **MAT** |
| --- | --- | --- | --- | --- | --- | --- |
| Nitrogen addition | **-0.0053*** | 0.0001 | 0.0011 | 0.0000 | -0.0002 | **-0.0271***** |
| Phosphorus addition | 0.0057 | - | - | **-0.0037***** | **0.0273***** | **-0.1097***** |
| Warming | -0.0050 | **0.0007*** | **-0.0351***** | **0.0001**** | **0.0009***** | -0.0143 |
| Increased precipitation | -0.0026 | 0.0000 | -0.0027 | 0.0000 | 0.0000 | -0.0040 |
| Reduced precipitation | 0.0029 | **0.0043***** | **-0.0417***** | **0.0001***** | -0.0004 | **-0.0345***** |
| Elevated CO_2_ | - | - | - | - | - | - |
| Grazing | -0.0119 | **-0.002***** | 0.0000 | **-0.0002***** | 0.0003 | **0.0313**** |
| vegetated soil | **0.0434***** | - | - | - | - | - |
| Elevated plant richness | **0.0228***** | - | - | - | - | - |
| Elevated litter richness | **0.0104**** | - | - | - | - | - |
| Home-field decomposition | 0.0070 | - | - | - | - | - |
| Soil biota exclusion | **0.0075***** | -0.0002 | -0.0004 | **-0.0001***** | **-0.0001***** | **-0.0144***** |

**Note:** Estimates (slope) are shown, and values in bold indicate significant effects. **P* < 0.05, ***P* < 0.01, ****P* < 0.001.

**Note：**The symbol ‘–’ denotes missing data or cases with fewer than three observations.

**Supplementary Table S2** Meta-regression analyses assessing the moderating effects of initial litter chemistry and elevated plant/litter richness on the effect sizes (logeRR) of root litter decomposition in response to environmental and biotic factors.

| **Treatment effects** | **C** | **N** | **P** | **C: N** | **N:P** | **Cellulose** | **AUR** | **AUR: N** | **Elevated Plant**  **/Litter richness** |
| --- | --- | --- | --- | --- | --- | --- | --- | --- | --- |
| Nitrogen addition | -0.0004 | 0.0024 | **-0.0878***** | **-0.0028*** | **0.0076*** | **-0.0003***** | **-0.0015***** | **-0.0130***** | - |
| Phosphorus addition | 0.0013 | 0.1696 | -1.6993 | -0.0136 | **0.0709*** | - | - | - | - |
| Warming | 0.0007 | -0.0160 | 0.0961 | 0.0006 | - | - | - | - | - |
| Increased precipitation | -0.0001 | 0.0014 | -0.0502 | 0.0008 | -0.0029 | - | 0.0004 | -0.0028 | - |
| Reduced precipitation | 0.0045 | 0.0000 | -0.7826 | 0.0027 | -0.0098 | **-0.0015*** | 0.0018 | -0.0021 | - |
| Elevated CO2 | - | - | - | - | - | - | - | - | - |
| Grazing | -0.0004 | -0.0053 | -0.0023 | 0.0019 | 0.0039 | **0.0012***** | -0.0011 | -0.0052 | - |
| vegetated soil | 0.0020 | **0.066**** | - | -0.0026 | - | -0.0011 | -0.0012 | -0.0195 | - |
| Elevated plant richness | - | - | - | - | - | - | - | - | **-0.0133***** |
| Elevated litter richness | - | - | - | - | - | - | - | - | **-0.0145***** |
| Home-field decomposition | 0.0003 | 0.0079 | -0.2137 | 0.0000 | -0.0223 | - | -0.0012 | 0.0002 | - |
| Soil biota exclusion | **-0.0041***** | **-0.0081*** | -0.0355 | -0.0008 | -0.0057 | - | - | - | - |

**Note:** Estimates (slope) are shown, and values in bold indicate significant effects. **P* < 0.05, ***P* < 0.01, ****P* < 0.001.

**Note:** The symbol ‘–’ denotes missing data or cases with fewer than three observations.
